# Muscle‐Specific Strength Better Predicts Physical Performance Decline Than Conventional Metrics: The I‐Lan Longitudinal Aging Study

**DOI:** 10.1002/jcsm.70078

**Published:** 2025-10-01

**Authors:** Wen‐Kai Chien, Wei‐Ju Lee, Chih‐Kuang Liang, Ko‐Han Yen, Li‐Ning Peng, Ming‐Hsien Lin, Ching‐Hui Loh, Fei‐Yuan Hsiao, Liang‐Kung Chen

**Affiliations:** ^1^ Institute of Hospital and Health Care Administration National Yang Ming Chiao Tung University Taipei Taiwan; ^2^ Center for Healthy Longevity and Aging Sciences National Yang Ming Chiao Tung University Taipei Taiwan; ^3^ Department of Family Medicine Taipei Veterans General Hospital Yuanshan Branch Yi‐Lan Taiwan; ^4^ Center for Geriatrics and Gerontology Kaohsiung Veterans General Hospital Kaohsiung Taiwan; ^5^ Center for Geriatrics and Gerontology Taipei Veterans General Hospital Taipei Taiwan; ^6^ Center of Health and Aging Hualien Tzu Chi Hospital Buddhist Tzu Chi Medical Foundation Hualien Taiwan; ^7^ Graduate Institute of Clinical Pharmacy National Taiwan University College of Medicine Taipei Taiwan; ^8^ Taipei Municipal Gan‐Dau Hospital Taipei Taiwan

**Keywords:** impaired physical performance, muscle quality index, muscle‐specific strength, sarcopenia

## Abstract

**Background:**

Muscle‐specific strength (MSS), defined as grip strength normalized to arm muscle mass, may better reflect muscle efficiency than grip strength. Despite its potential utility in diagnosing sarcopenia, longitudinal studies evaluating MSS as a predictor of sarcopenia‐related outcomes remain limited. This study investigated the associations of MSS with physical performance deterioration and biomarker profiles in community‐dwelling older adults, compared it with conventional measures, determined whether MSS‐defined sarcopenia offers superior predictive utility versus traditional diagnostic criteria and examined associations with key cardiometabolic and inflammatory biomarkers.

**Methods:**

This prospective cohort study included 1609 participants (mean age 64.5 ± 6.7 years; 50.5% male) from the I‐Lan Longitudinal Aging Study. MSS was calculated by dividing handgrip strength by dominant arm muscle mass. Participants were categorized into low and normal MSS groups using age‐sex‐specific cutoffs based on quintiles. Physical function was assessed using the five‐time chair stand test, with impaired physical performance (PPI) defined as taking ≥ 12 s. Logistic regression models examined the associations between MSS and PPI, adjusted for demographics, comorbidities, cognitive function and skeletal muscle index. Biomarker profiles, including metabolic, inflammatory and hormonal parameters, were compared across MSS groups.

**Results:**

Low MSS was found in 19.9% of participants. Those with low MSS had significantly higher muscle mass (skeletal muscle index, 7.7 ± 1.2 vs. 7.1 ± 1.2 kg/m^2^, *p* < 0.001; dominant hand muscle mass, 2.7 ± 0.8 vs. 2.3 ± 0.6 kg, *p* < 0.001) but weaker grip strength (26.1 ± 8.1 vs. 32.0 ± 8.8 kg, p < 0.001), indicating disproportionately low strength relative to muscle size. PPI was more common in the low MSS group (47.8% vs. 29.0%, *p* < 0.001). Low MSS was significantly associated with higher odds of PPI (adjusted OR = 1.49, 95% CI: 1.11–1.99, *p* = 0.008), particularly among participants aged ≥ 65 years (OR = 1.80, 95% CI: 1.18–2.74, *p* = 0.006) and males (OR = 1.64, 95% CI: 1.09–2.47, *p* = 0.018). MSS‐defined sarcopenia showed a stronger association with PPI (OR = 3.31, 95% CI: 1.26–8.74, *p* = 0.015) than conventional sarcopenia definitions. Individuals with low MSS demonstrated adverse metabolic profiles, including higher fasting glucose (101.0 ± 27.3 vs. 95.0 ± 18.7 mg/dL, *p* < 0.001), HbA1c (6.0% ± 0.9% vs. 5.8% ± 0.7%, *p* < 0.001) and HOMA‐IR (2.6 ± 2.2 vs. 1.8 ± 1.4, *p* < 0.001). In adjusted models, high HOMA‐IR (OR = 2.71, 95% CI: 1.94–3.79, *p* < 0.001) and elevated hsCRP (OR = 1.66, 95% CI: 1.18–2.33, *p* = 0.001) were strongly associated with low MSS.

**Conclusions:**

Low MSS independently predicts physical performance deterioration and associates with adverse metabolic and inflammatory biomarker profiles in older adults. MSS may better indicate muscle health than traditional metrics, supporting its inclusion in sarcopenia assessment frameworks as recommended by recent consensus guidelines.

## 1 Introduction

Sarcopenia, characterized by the age‐related progressive loss of muscle mass and function, significantly increases the risk of impaired physical performance, disability, reduced quality of life and mortality among older adults [1, 2]. Previously, sarcopenia assessment has relied on separate measurements of muscle mass or function [3]. Yet, taken alone, these indicators often fall short in capturing overall muscle function. Therefore, current consensus definitions of sarcopenia, including those from the European Working Group on Sarcopenia in Older People (EWGSOP2) and the Asian Working Group for Sarcopenia (AWGS 2019), emphasize combined muscle mass, muscle strength and physical performance as the diagnostic components [4, 5]. Recently, the Global Leadership Initiative on Sarcopenia (GLIS) has proposed a new diagnostic algorithm for sarcopenia that designates muscle strength or muscle‐specific strength (MSS) as the primary metric for assessing muscle function, while reclassifying physical performance parameters as outcome indicators of sarcopenic progression [6].

In addition to muscle strength, some researchers use the Muscle Quality Index (MQI)—grip strength divided by total appendicular lean mass—as a marker of muscle efficiency [7, 8]. However, MQI has inherent limitations related to sex‐specific differences in muscle distribution and potential misclassification due to differences in body composition. Men typically carry relatively more muscle mass in the upper body, whereas women have a higher proportion of lean mass concentrated in the lower limbs [9]. Moreover, when grip strength is normalized to total lean mass, obese individuals may appear to have artificially low MQI values due to high leg mass inflating the denominator, despite having adequate upper limb strength.

MSS, defined as grip strength normalized specifically to upper limb lean mass, reduces the biases derived from MQI. Despite the potential utility of MSS in diagnosing sarcopenia, longitudinal studies evaluating MSS as a predictor of sarcopenia‐related outcomes remain scarce, particularly among Asian populations, thus limiting the clinical application of MSS as a diagnostic criterion for sarcopenia. This current study aimed to evaluate MSS's prognostic value for physical performance deterioration in community‐dwelling older adults, compare it with conventional measures (grip strength and MQI), determine whether MSS‐defined sarcopenia offers superior predictive utility versus traditional diagnostic criteria and examine associations between MSS and key cardiometabolic and inflammatory biomarkers.

## 2 Methods

### 2.1 Participants and Study Design

**FIGURE 1:**
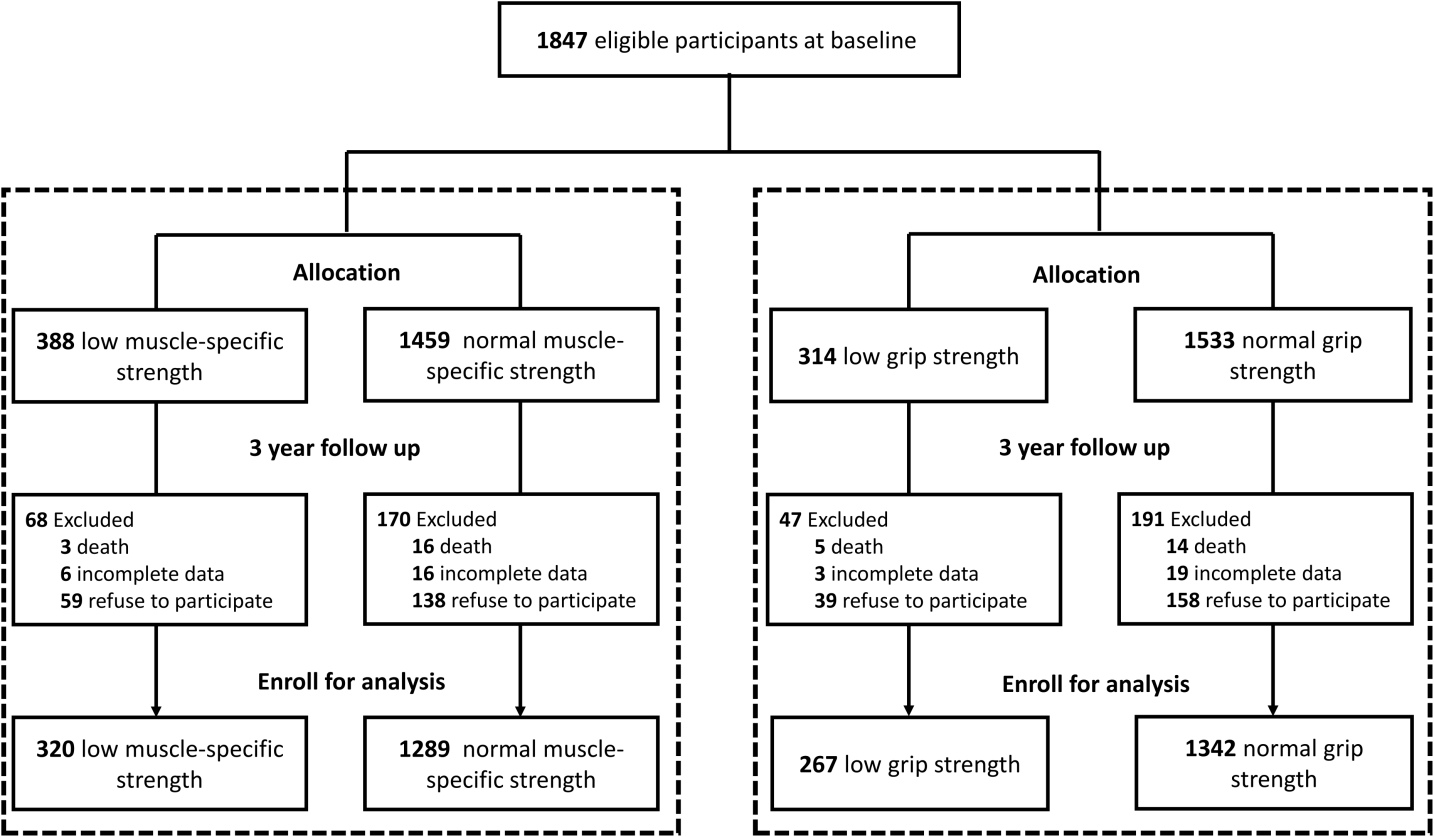
Participant recruitment flowchart.

The I‐Lan Longitudinal Aging Study (ILAS), initiated in 2011, is a prospective cohort study examining sarcopenia, frailty and cognitive function across ageing in community‐dwelling middle‐aged and older adults. Comprehensive descriptions of the study design and participant recruitment methods have been published previously [10, 11, 12]. Briefly, ILAS enrolled individuals aged 50 years or older who reside in Yuanshan Township, Yilan, Taiwan. ILAS recruited community residents aged 50 or older, excluding those unable to cooperate or communicate with researchers, unwilling to provide informed consent, institutionalized, functionally dependent or expected to live less than 6 months. For the current study, data from the third and fourth waves of ILAS were retrieved for analysis. Of the original 2020 community‐dwelling older adults who underwent face‐to‐face interviews in 2020, 173 were excluded due to incomplete Bioelectrical Impedance Analysis (BIA) data in Wave 3. Besides, 19 participants were lost to follow‐up due to death, 22 due to incomplete data and 197 reluctant to participate. A total of 1609 participants were included in the final analysis (Figure 1).

The institutional review board of Taipei Veterans General Hospital (2018‐05‐003B) approved the study protocol, and written informed consent was obtained from each individual before inclusion. This study was designed and conducted in accordance with the principles of the 1964 Declaration of Helsinki and later amendments. Reporting of this study adhered to the Strengthening the Reporting of Observational Studies in Epidemiology (STROBE) guidelines [13].

### 2.2 Muscle Mass Measurement

Body composition was assessed using multifrequency BIA (Inbody S10, Seoul, South Korea), measuring lean body mass, estimated appendicular muscle mass and lean muscle mass of the dominant hand. Appendicular skeletal muscle mass (ASM) was derived by summing lean tissue mass from all four limbs, and skeletal muscle index was calculated as ASM divided by height squared (kg/m^2^). Low muscle mass was defined as a height‐adjusted skeletal muscle index below 7 kg/m^2^ for men and below 5.7 kg/m^2^ for women aged 65 years or older and below the lowest quintile thresholds (7.8 kg/m^2^ for men and 5.7 kg/m^2^ for women) for those younger than 65 years [5].

### 2.3 Definition of Grip Strength, Muscle‐Specific Strength, Muscle Quality Index and Sarcopenia

Hand grip strength (HGS) of the dominant hand was measured using a digital dynamometer (Smedley's Dynamo Meter; TTM, Tokyo, Japan), and the highest value among three trials was used for statistical analysis. MSS was calculated by dividing HGS by the lean muscle mass of the dominant hand. The MQI was defined as HGS divided by ASM.

Low MSS and low MQI were defined as values within the lowest quintile, stratified by sex and age group (< 65 years and ≥ 65 years). Low MSS was defined as < 11.9 kg/kg for males and < 10.9 kg/kg for females aged < 65 years and < 11.0 kg/kg for males and < 10.6 kg/kg for females aged ≥ 65 years. Low MQI was defined as < 1.45 kg/kg for males and < 1.39 kg/kg for females aged < 65 years and < 1.38 kg/kg for both sexes aged ≥ 65 years. Low HGS was defined as < 28 kg for men and < 18 kg for women aged 65 years or older and below the lowest quintile thresholds (< 33.8 kg for men and < 21.4 kg for women) for those younger than 65 years [5].

Sarcopenia was defined as the coexistence of low muscle strength and low muscle mass. Based on the specific definitions of muscle strength, we further categorized sarcopenia into three subtypes: (1) HGS‐sarcopenia, defined as low HGS plus low muscle mass; (2) MSS‐sarcopenia, defined as low MSS plus low muscle mass; and (3) MQI‐sarcopenia, defined as low MQI plus low muscle mass.

### 2.4 Outcome Measurement

GLIS suggests that physical performance should be considered an optimal health outcome rather than a diagnostic component of sarcopenia, given its susceptibility to influence by neuronal and other nonmuscular factors [14]. Therefore, we selected physical performance as the primary outcome in this study. Impaired physical performance was defined as taking 12 s or more to complete five chair stand repetitions, in accordance with the criteria from AWGS 2019 and GLIS [5, 6].

### 2.5 Functional Assessment

Research nurses collected demographic data, anthropometric measurements and functional assessments through face‐to‐face interviews. The collected information included age, sex, educational level, smoking and drinking habits. Standardized assessments were also conducted, including the Mini‐Nutritional Assessment (MNA) to evaluate nutritional status [15], the Center for Epidemiological Studies Depression Scale (CES‐D) to assess depressive symptoms [16] and the Mini‐Mental State Examination (MMSE) to evaluate cognitive function [17]. In addition, the Functional Autonomy Measurement System (SMAF) was used to assess activities of daily living (ADL) and instrumental activities of daily living (IADL) [18], and the Charlson Comorbidity Index (CCI) was applied to quantify disease burden [19].

### 2.6 Laboratory Data

Peripheral venous blood samples were collected after a 10‐h overnight fast. A broad range of biomarkers was analysed to assess cardiometabolic health, hormonal profiles and inflammatory status. These biomarkers included fasting glucose, glycated haemoglobin, total cholesterol, triglycerides, low‐density lipoprotein (LDL), insulin resistance estimated by the homeostasis model assessment (HOMA‐IR) and insulin secretory capacity assessed by the homeostatic model assessment of β‐cell function HOMA‐β [20]. Inflammatory markers such as neutrophil‐to‐lymphocyte ratio (NLR), platelet‐to‐lymphocyte ratio, homocysteine and high‐sensitivity C‐reactive protein (hsCRP) were also measured. Levels of age‐related hormones, including insulin‐like growth factor 1 (IGF‐1), and micronutrients such as vitamin D (25‐hydroxyvitamin D) were assessed. Technical specifications, including assay equipment and intraassay and interassay coefficients of variation, are detailed in Table S1.

### 2.7 Statistical Analysis

In this study, continuous variables were presented as means with standard deviations, and categorical variables were summarized as counts and percentages. Descriptive statistics were compared using Student's *t* test for continuous variables and chi‐square or Fisher's exact test for categorical variables, as appropriate.

MSS, HGS, MQI and sarcopenia defined by low strength, low MSS or low MQI were treated as categorical variables to assess their associations with impaired physical performance through logistic regression analysis. Multinomial logistic regression was employed to examine associations between MSS and baseline biomarker tertiles. Inverse probability weighting was applied as a sensitivity analysis to handle missing data. Subgroup analyses were conducted based on age (< 65 vs. ≥ 65 years), sex (male vs. female), diabetes mellitus (yes vs. no) and hypertension (yes vs. no). We conducted additional analyses to account for adiposity by incorporating models of sarcopenic obesity and MSS‐defined sarcopenic obesity. Sarcopenic obesity was defined as the coexistence of low muscle mass and excessive adiposity, based on criteria from previous studies [12]. We also performed multivariable logistic regression including MSS and body fat percentage simultaneously to evaluate their independent associations with impaired physical performance. For all tests, statistical significance was defined as a two‐sided *p* value < 0.05 and a 95% confidence interval that did not include the null value. All statistical analyses were performed using SAS software, Version 9.4 (SAS Institute Inc., Cary, NC, United States).

## 3 Results

### 3.1 Baseline Characteristics of Study Participants

A total of 1609 participants were included, with 320 (19.9%) classified as having low MSS (Table 1). During the follow‐up period, 731 participants (45.4%) experienced physical performance decline. Participants with low MSS had significantly higher muscle mass (skeletal muscle index, 7.7 ± 1.2 kg/m^2^ vs. 7.1 ± 1.2 kg/m^2^, *p* < 0.001, and dominant hand muscle mass, 2.7 ± 0.8 kg vs. 2.3 ± 0.6 kg, p < 0.001) but weaker grip strength (26.1 ± 8.1 kg vs. 32.0 ± 8.8 kg, p < 0.001), indicating disproportionately low strength relative to muscle size. Individuals in the low MSS group exhibited poorer cognitive function (MMSE, 26.9 ± 3.8 vs. 27.9 ± 2.5, *p* < 0.001), higher body mass index and higher percentages of hypertension (41.9% vs. 29.3%, *p* < 0.001) and diabetes (20.9% vs. 12.8%, *p* < 0.001). No significant differences were observed in age, nutritional status, depressive symptoms, functional activities, comorbidity burden or other chronic conditions (Table 1).

**TABLE 1:** Baseline characteristics of participants stratified by muscle strength.

Characteristics: Data show mean ± standard deviation or number (%)	Muscle specific strength (MSS)			Hand grip strength (HGS)		
	Low MSS (*n* = 320)	Normal MSS (*n* = 1289)	*p* value	Low HGS (*n* = 267)	Normal HGS (*n* = 1342)	*p* value
Demographic and lifestyle factors						
Age (years)	65.4 ± 7.8	64.5 ± 6.7	0.055	65.1 ± 7.8	64.6 ± 0.2	0.266
Male	162 (50.6)	650 (50.5)	1	133 (49.8)	680 (50.7)	0.841
Education level			< 0.001			0.784
Below elementary school	107 (33.4)	288 (22.3)		70 (26.2)	325 (24.2)	
Junior high school	50 (15.6)	205 (15.9)		41 (15.4)	214 (16.0)	
Above high school	163 (50.9)	796 (61.8)		156 (58.4)	803 (59.8)	
Current alcohol	76 (23.8)	311 (24.1)	0.942	55 (20.6)	332 (24.7)	0.159
Current tobacco smoker	35 (10.9)	107 (8.3)	0.152	29 (10.6)	113 (8.4)	0.195
Body mass index (kg/m^2^)	26.4 ± 3.7	23.9 ± 3.0	< 0.001	23.8 ± 3.2	24.5 ± 3.3	0.003
Medical history						
Hypertension	134 (41.9)	377 (29.3)	< 0.001	85 (31.8)	426 (31.7)	1
Diabetes	67 (20.9)	165 (12.8)	< 0.001	37 (22.8)	90 (13.8)	0.007
Coronary artery disease	12 (3.8)	59 (4.6)	0.648	9 (3.4)	62 (4.6)	0.418
Stroke	1 (0.3)	15 (1.2)	0.221	2 (0.8)	14 (1.0)	1
COPD	3 (0.9)	4 (0.3)	0.146	0	7 (0.5)	0.608
Chronic kidney disease	3 (0.9)	6 (0.5)	0.394	0	9 (0.7)	0.371
Charlson comorbidity index	0.3 ± 0.5	0.2 ± 0.5	0.123	0.2 ± 0.5	0.2 ± 0.5	0.683
Functional assessments						
Mini‐Mental State Examination	26.9 ± 3.8	27.9 ± 2.5	< 0.001	27.1 ± 3.5	27.8 ± 2.7	0.001
Mini‐Nutrition Assessment	27.3 ± 1.8	27.3 ± 1.7	0.468	26.7 ± 2.0	27.4 ± 1.6	< 0.001
Center for Epidemiological Studies Depression Scale	1.1 ± 2.5	1.4 ± 3.8	0.076	1.6 ± 3.7	1.2 ± 3.5	0.108
SMAF‐ADL	−0.02 ± 0.28	−0.01 ± 0.23	0.680	−0.02 ± 0.31	−0.01 ± 0.23	0.574
SMAF‐IADL	−0.09 ± 0.77	−0.05 ± 0.68	0.419	−0.13 ± 0.93	−0.04 ± 0.64	0.138
Muscle health parameters						
Muscle‐specific strength	9.7 ± 1.3	14.3 ± 2.3	< 0.001	11.0 ± 2.3	13.9 ± 2.6	< 0.001
Grip strength (kg)	26.1 ± 8.1	32.0 ± 8.8	< 0.001	23.0 ± 6.2	32.4 ± 8.6	< 0.001
Skeletal muscle index (kg/m^2^)	7.7 ± 1.2	7.1 ± 1.2	< 0.001	7.1 ± 1.1	7.3 ± 1.2	0.006
Dominant hand muscle mass (kg)	2.7 ± 0.8	2.3 ± 0.6	< 0.001	2.1 ± 0.5	2.4 ± 0.7	< 0.001
Impaired physical performance	153 (47.8)	374 (29.0)	< 0.001	111 (41.6)	416 (31.0)	0.001

Abbreviations: ADL, activities of daily living; HGS, hand grip strength; IADL, instrumental activities of daily living; MSS, muscle‐specific strength; SMAF, functional autonomy measurement system.

Table S2 compares baseline characteristics across participants classified using three sarcopenia definitions: MSS‐sarcopenia, HGS‐based sarcopenia and MQI‐sarcopenia. Participants with MSS‐sarcopenia had significantly lower BMI, poorer nutritional status (MNA score) and reduced muscle mass and strength compared to those without MSS‐sarcopenia. MQI‐sarcopenia and HGS‐based sarcopenia shared similar trends to MSS‐sarcopenia but identified fewer individuals.

### 3.2 Association of MSS and Sarcopenia With Adverse Outcomes

**FIGURE 2:**
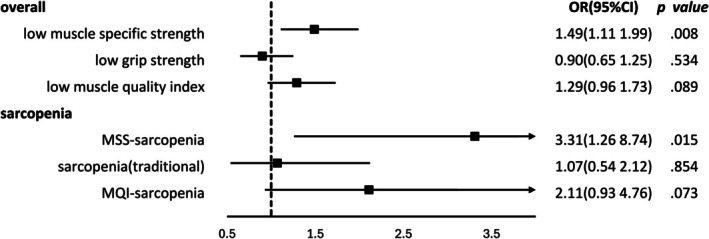
Impact of different strengths and different types of sarcopenia on physical functional impairment.

Full adjusted multiple logistic regression analyses explored the relationship between MSS and impaired physical performance (Figure 2). The findings indicate that low MSS is associated with a 49% increased risk of impaired physical performance (*p* = 0.008), whereas low HGS and low MQI were not significantly associated. MSS‐sarcopenia was significantly associated with impaired physical performance (OR 3.31, 95% CI: 1.26–8.74, *p* = 0.015), whereas MQI‐sarcopenia and HGS‐sarcopenia were not statistically significant (Figure 2 and Table S3). Results from the sensitivity analysis using inverse probability weighting to account for missing follow‐up data were consistent with the primary findings (Figure S1).

### 3.3 Subgroup Analysis of MSS and Impaired Physical Performance

**FIGURE 3:**
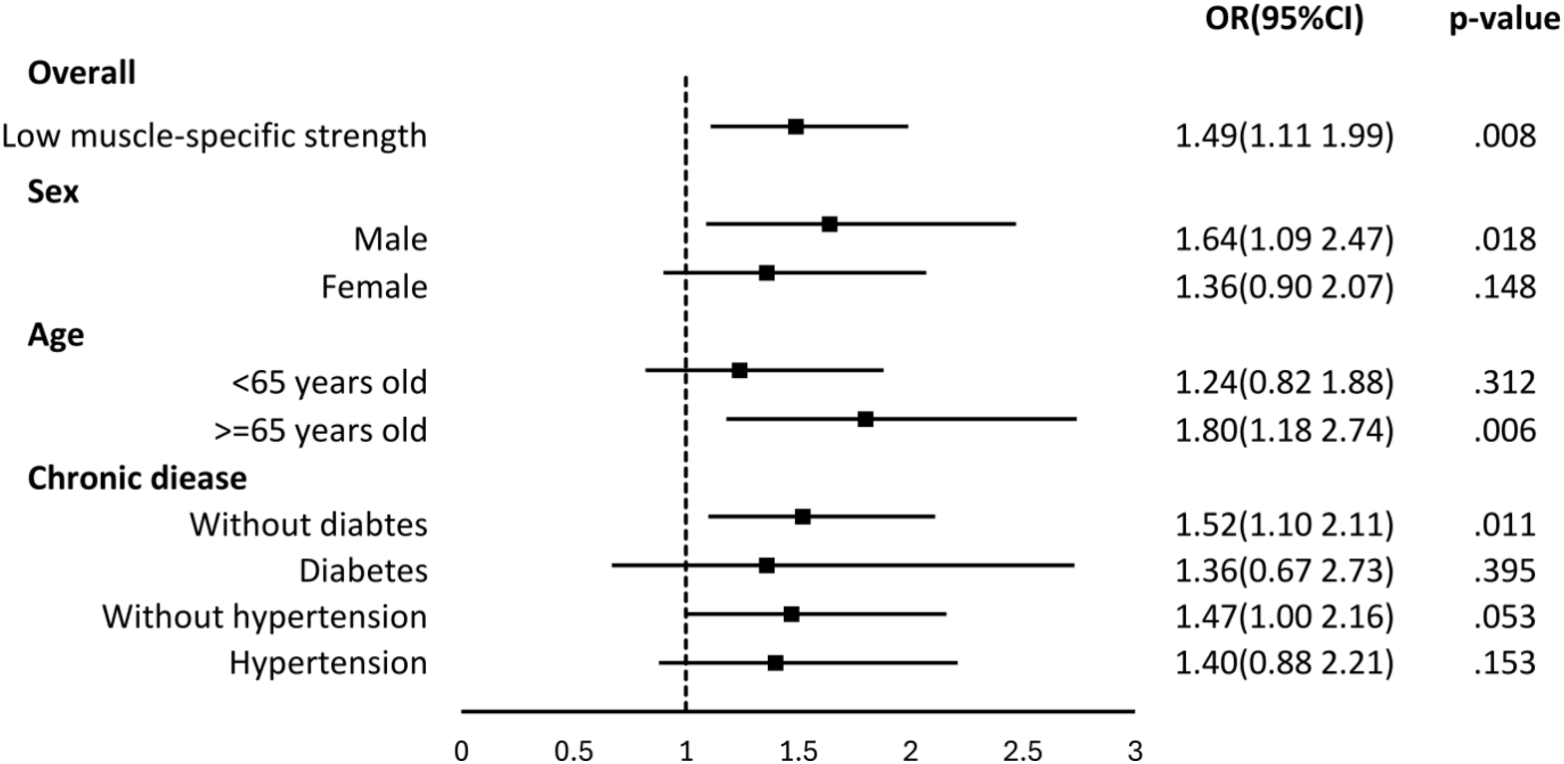
Impact of muscle‐specific strength subgroup on physical performance impairment.

In the subgroup analysis, low MSS was significantly associated with impaired physical performance in men (OR = 1.64, 95% CI: 1.09–2.47, *p* = 0.018), adults aged ≥ 65 years (OR = 1.80, 95% CI: 1.18–2.74, *p* = 0.006) and individuals without diabetes (OR = 1.52, 95% CI: 1.10–2.11, *p* = 0.011). However, no significant associations were found among women, participants under 65 years of age or individuals with or without hypertension and with diabetes (Figure 3 and Table S4).

Both sarcopenic obesity and MSS‐defined sarcopenic obesity were significantly associated with higher odds of impaired physical performance (Figure S2). In models simultaneously adjusting for MSS and body fat percentage, MSS remained independently associated with impaired physical performance (adjusted OR = 1.36, 95% CI: 1.01–1.83, *p* = 0.041), indicating its predictive value beyond adiposity.

### 3.4 Association of Muscle‐Specific Strength With Biomarkers

**FIGURE 4:**
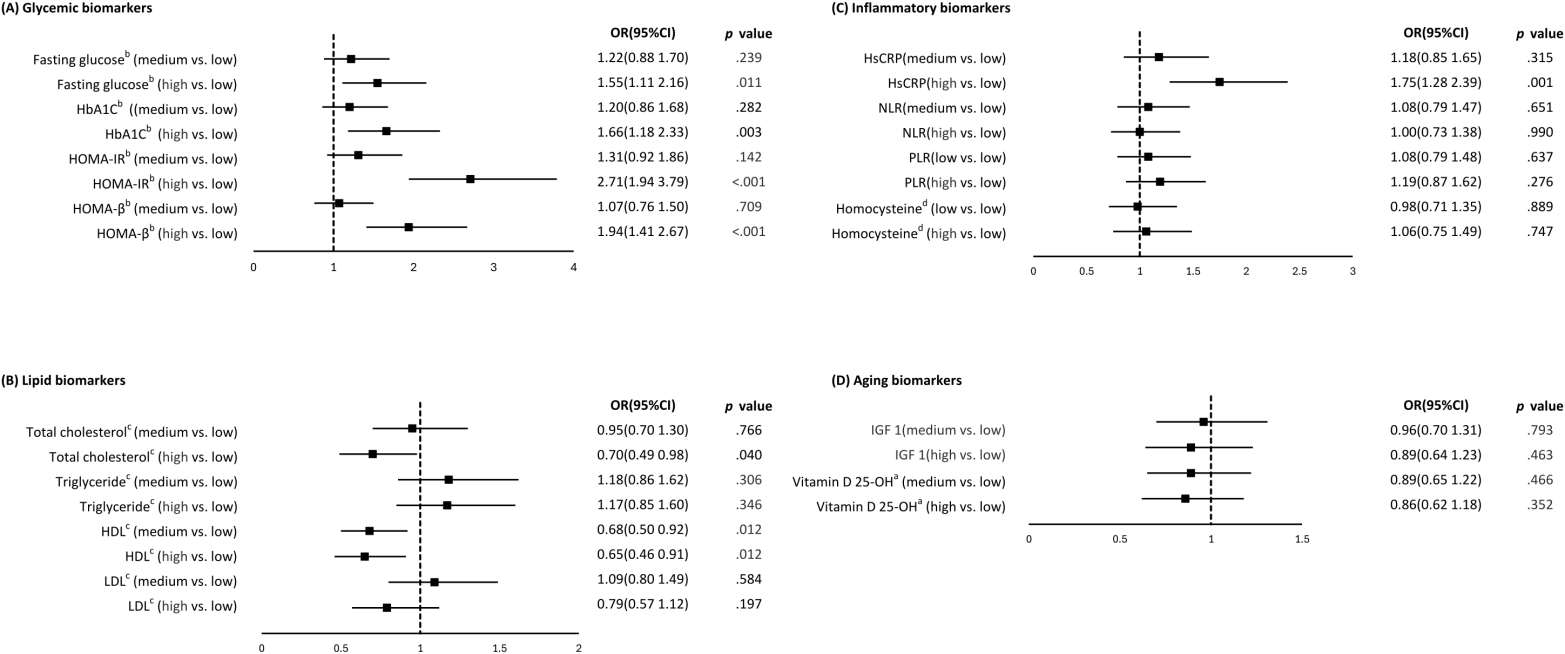
Multinomial logistic regressions explore associations between muscle‐specific strength and biomarkers.

Participants with low MSS had significantly higher levels of fasting glucose, HbA1c, HOMA‐IR, triglycerides and LDL‐C, alongside lower levels of HDL‐C and total cholesterol (TC), indicating a more adverse cardiometabolic profile. No significant differences were observed in ageing‐related biomarkers (IGF‐1, vitamin D) or most inflammatory markers, except for a trend towards higher hsCRP levels (Table S5). Cardiometabolic risk factors and chronic inflammatory biomarkers were dichotomized into tertiles to examine the associations between baseline biomarker levels and low MSS. Full adjusted multinomial logistic regression showed that higher fasting glucose was significantly associated with low MSS (OR = 1.55, 95% CI: 1.11–2.16, *p* = 0.011). Similarly, elevated HbA1c (OR = 1.66, 95% CI: 1.18–2.33, *p* = 0.003) and increased insulin resistance, as measured by HOMA‐IR (OR = 2.71, 95% CI: 1.94–3.79, *p* < 0.001), were also significantly associated with low MSS. HOMA‐β showed a negative association with MSS, with lower insulin secretory capacity linked to higher odds of low MSS (OR = 1.94, 95% CI: 1.41–2.67, *p* < 0.001). Additionally, higher TC (OR = 0.70, 95% CI: 0.49–0.98, *p* = 0.040) and elevated HDL (OR = 0.65, 95% CI: 0.46–0.91, *p* = 0.012) were also linked to low MSS. Among chronic inflammatory biomarkers, hsCRP showed a positive association with low MSS (OR = 1.75, 95% CI: 1.28–2.39, *p* = 0.001) (Figure 4 and Table S5).

## 4 Discussion

This study demonstrates that MSS, calculated as grip strength adjusted by the lean muscle mass of the dominant hand, predicts impaired physical performance over a 3‐year follow‐up more effectively than HGS or MQI. When combined with low muscle mass, MSS‐defined sarcopenia showed a stronger predictive value than traditional definitions of sarcopenia. This highlights the potential of MSS as an early clinical marker of muscle function, in line with the recommendations of the GLIS. Subgroup analyses revealed a higher risk among older adults and males, likely reflecting sex‐specific physiological differences and age‐related disparities in the rate of muscle decline. Furthermore, significant associations between MSS and cardiometabolic and inflammatory markers—such as fasting glucose, HOMA‐IR and hsCRP—support metabolic dysregulation and chronic inflammation as underlying pathophysiological mechanisms affecting muscle health [21].

The recent Delphi consensus from the GLIS has recognized MSS—defined as strength normalized to muscle size—as a diagnostic component of sarcopenia, alongside muscle mass and strength [6]. This landmark consensus reflects growing recognition that sarcopenia is not solely a disorder of reduced muscle quantity but also of diminished functional capacity relative to muscle size. Our study provides timely and robust empirical support for prioritizing MSS in the sarcopenia diagnosis and extends its clinical relevance by demonstrating MSS as a practical, performance‐linked and biologically meaningful metric. MSS integrates both functional and structural dimensions of skeletal muscle by normalizing strength measurements to muscle mass, thereby quantifying contractile efficiency and facilitating identification of individuals with preserved muscle mass but diminished functional capacity. Furthermore, MSS‐based sarcopenia was more predictive of functional impairment than conventional sarcopenia definitions or MQI‐sarcopenia. These findings underscore the potential of MSS to serve as a more sensitive and specific indicator for sarcopenia diagnosis.

Muscle strength declines much earlier than muscle mass with age, leading to a mismatch that becomes more pronounced over time [22, 23]. This discrepancy underscores the clinical relevance of MSS, a metric that reflects the efficiency of force generation relative to muscle size. Our study found that low MSS was significantly associated with impaired physical performance in older adults but not in middle‐aged individuals. This age‐specific pattern suggests that MSS better captures ageing‐related neuromuscular deterioration that is not yet apparent in midlife. The stronger association between MSS and poor physical function in older adults may reflect physiological changes such as reduced motor unit recruitment, loss of maximal oxygen consumption and shifts in muscle composition [24]. These changes likely diminish the ability of older adults to compensate for declining muscle composition, making MSS a more sensitive indicator of early vulnerability. In contrast, middle‐aged individuals may still possess sufficient muscle reserve and functional adaptability to mask subtle declines in muscle quality. Our findings support that MSS may serve as an early marker of functional impairment risk, identifying older adults with hidden muscle inefficiency despite normal mass or strength.

Sex‐specific differences in muscle biology and body composition necessitate separate analyses in sarcopenia research to improve understanding and intervention strategies. Haizlip et al. [25] described intrinsic sex differences in muscle fibre composition and contractility, which may partly explain divergent patterns in muscle ageing. This distinction is particularly important in Asian populations, where women tend to present higher adiposity and smaller body sizes than men [23], making them susceptible to sarcopenia via different mechanisms. Biomarker evidence further supports sex‐dependent mechanisms. Myostatin, a negative regulator of muscle growth, has been associated with low skeletal muscle mass exclusively in men [26]. A recent review demonstrated that exercise‐induced activation of muscle stem cells occurs more prominently in men than in women [27]. Results of our study show that low MSS is associated with impaired physical performance in both sexes, with stronger effects in men. These observations underline the importance of considering sex‐specific biological and environmental factors in both research and clinical management of sarcopenia. Moreover, several cardiometabolic and inflammatory biomarkers have been found to be significantly associated with MSS. Elevated markers of glucose metabolism (fasting glucose, HbA1c and HOMA‐IR) and lipid profile as increased TC and reduced HDL‐C were consistently observed among individuals with lower MSS. This finding was similar to the previous study, which reported a strong link between relative muscle strength and cardiometabolic profiles [28]. Furthermore, a recent omics‐based analysis reinforces the critical role of glucose dysregulation in mobility impairment, implicating metabolic shifts as potential drivers of muscle decline [29]. HOMA‐β was negatively associated with MSS. This relationship may be partly mediated by the inverse association between HOMA‐β and BMI, as individuals in the low MSS group exhibited significantly higher BMI [30]. Systemic low‐grade inflammation also emerged as a relevant factor. Higher levels of hsCRP were significantly linked with low MSS, supporting the concept of ‘inflammaging’—the chronic, smouldering inflammation that contributes to age‐related functional decline [31]. Even in a relatively healthy older population, elevated hsCRP may signal early inflammatory processes detrimental to muscle function. Taken together, these biomarker profiles reflect interconnected physiological disturbances—metabolic and inflammatory—that may contribute to the pathogenesis of muscle weakness.

Despite all the efforts carried out in the study, several limitations should be acknowledged. First, the study participants were relatively healthy community‐dwelling adults, which may have led to higher baseline physical performance and underestimated the predictive value of MSS. Second, the follow‐up duration of approximately 3 years may not have been sufficient to detect longer‐term declines in physical function. Future studies with extended follow‐up are warranted to validate our findings. Third, although BIA is less accurate than gold‐standard imaging modalities such as CT or MRI, it remains a practical method for community‐based research due to its portability, low cost and ease of use [32]. Previous studies have demonstrated its reasonable validity when compared to dual‐energy X‐ray absorptiometry (DXA) [33]. In particular, DXA exhibits limited capacity to distinguish intramuscular adipose tissue from lean mass, potentially resulting in muscle mass overestimation, especially among Asian female populations who demonstrate higher adiposity profiles. Despite the aforementioned limitations, this study demonstrates significant merit as the first prospective cohort study in Asia to compare different standardization approaches for HGS in predicting subsequent functional decline, providing critical insights for sarcopenia diagnosis, suggesting that MSS may warrant prioritization over absolute strength measurements.

In conclusion, this study highlights MSS—grip strength normalized to dominant hand muscle mass—as a sensitive and effective marker of impaired physical performance. MSS outperformed conventional measures and showed a stronger link to impaired function than HGS‐ and MQI‐defined sarcopenia. Its association with unfavourable cardiometabolic and inflammatory profiles supports MSS as a biologically plausible indicator of muscle health. Given its simplicity and predictive value, MSS may be a useful tool for early detection and monitoring of muscle health in ageing.

## Conflicts of Interest

The authors declare no conflicts of interest.

## Supporting information


**Table S1:** Details of measurement devices for serum biomarkers.
**Table S2:** Baseline characteristics of participants stratified by sarcopenia defined by muscle specific strength, grip strength and muscle quality index.
**Table S3:** Associations of muscle‐specific strength, grip strength, muscle quality index, and sarcopenia with impaired physical performance.
**Table S4:** Muscle‐specific strength and impaired physical performance stratified by age, sex and chronic conditions.
**Table S5:** Baseline biomarker profile stratified by muscle specific strength.
**Table S6:** Multinomial logistic regressions explore associations between muscle‐specific strength and biomarkers.
**Figure S1:** Sensitivity analysis using inverse probability weighting to account for missing follow‐up data.
**Figure S2:** Associations of MSS, sarcopenic obesity, and MSS‐sarcopenic obesity with impaired physical performance.
