# Supplementary material for: Muscle‐Specific Strength Better Predicts Physical Performance Decline Than Conventional Metrics: The I‐Lan Longitudinal Aging Study
Source: J Cachexia Sarcopenia Muscle. 2025 Oct 1;16(5):e70078. doi: 10.1002/jcsm.70078 (PMC12485295; doi:10.1002/jcsm.70078)
Supplement: Supplementary file 1 — Table S1: Details of measurement devices for serum biomarkers. Table S2: Baseline characteristics of participants stratified by sarcopenia defined by muscle specific strength, grip strength and muscle quality index. Table S3: Associations of muscle‐specific strength, grip strength, muscle quality index, and sarcopenia with impaired physical performance. Table S4: Muscle‐specific strength and impaired physical performance stratified by age, sex and chronic conditions. Table S5: Baseline biomarker profile stratified by muscle specific strength. Table S6: Multinomial logistic regressions explore associations between muscle‐specific strength and biomarkers. Figure S1: Sensitivity analysis using inverse probability weighting to account for missing follow‐up data. Figure S2: Associations of MSS, sarcopenic obesity, and MSS‐sarcopenic obesity with impaired physical performance. [file JCSM-16-e70078-s001.docx]

**Supplementary Material**

**Supplementary Table S1.** Details of measurement devices for serum biomarkers……………………………………………………………………………………………………….……...2

**Supplementary Table S2.** Table S2. Baseline characteristics of participants stratified by sarcopenia defined by muscle specific strength, grip strength and muscle quality index………………………………………………………………………………………………………………………..4

**Supplementary Table S3.** Associations of muscle-specific strength, grip strength, muscle quality index, and sarcopenia with impaired physical performance.……………………………………………………………………………………………………………5

**Supplementary Table S4.** Muscle-specific strength and impaired physical performance stratified by age, sex and chronic conditions………………………………………………………………………………………………………………..6

**Supplementary Table S5.** Baseline biomarker profile stratified by muscle specific strength……………………………………………………………………………………………………………………7

**Supplementary Table S6.**Multinomial logistic regressions explore associations between muscle-specific strength and biomarkers………………………………………………………………….……………………………………………9

**Supplementary figure S1.** Sensitivity analysis using inverse probability weighting to account for missing follow-up data……………….………………………………………….………….12

**Supplementary Figure S2.** Associations of MSS, sarcopenic obesity, and MSS-sarcopenic obesity with impaired physical performance…………………….………………..13

| **Supplementary Table S1.** Details of measurement devices for serum biomarkers | | | | | | | |
| --- | --- | --- | --- | --- | --- | --- | --- |
| Biomarkers | Unit | Methods | Machine | Company | Country | Intra- assay coefficients of variation | Inter-assay coefficients of variation |
| Fasting glucose | mg/dL | Hexokinase | ADVIA Chemistry XPT | SIEMENS | Germany | 1.46 | 1.19 |
| Glycated hemoglobin | % | HPLC | Bio-Rad D-100 System | Bio-Rad | USA | 1.03 | 0.85 |
| Insulin | mU/L | Chemiluminescence | ADVIA CentaurXPT | SIEMENS | Germany | 4.79 | 4.17 |
| Cholesterol | mg/dL | Enzymatic | ADVIA Chemistry XPT | SIEMENS | Germany | 1.51 | 0.92 |
| Triglycerides | mg/dL | Enzymatic GPO | ADVIA Chemistry XPT | SIEMENS | Germany | 2 | 1.22 |
| HDL | mg/dL | Direct Homogenous Surfactant | ADVIA Chemistry XPT | SIEMENS | Germany | 3.44 | 0.98 |
| LDL | mg/dL | Direct Homogenous Surfactant | ADVIA Chemistry XPT | SIEMENS | Germany | 2.54 | 1.68 |
| Homocysteine | μmol/L | Chemiluminescence | ADVIA CentaurXPT | SIEMENS | Germany | 5.52 | 5.24 |
| IGF-1 | ng/mL | Chemiluminescence Immunoassay | DOC Immulite 2000 Xpi | SIEMENS | Germany | 5.11 | 5.73 |
| hsCRP | mg/dL | Latex enhanced immunoturbidimetric | ADVIA Chemistry XPT | SIEMENS | Germany | 2.898 | 1.251 |
| White blood cell | 10^3^/μL | Microscopy | XN-9000 | SYSMEX | Japan | 1.21 | 1.19 |
| Platelet | 10³/μL | Microscopy | XN-9000 | SYSMEX | Japan | 2.96 | 1.09 |
| Neutrophil | % | Microscopy | XN-9000 | SYSMEX | Japan | 2.36 | 1.24 |
| Lymphocyte | % | Microscopy | XN-9000 | SYSMEX | Japan | 3.36 | 1.76 |
| 25-OH vitamin D | ng/mL | Chemiluminescence | LIAISON | DiaSorin | Italy | 3.77 | 4.01 |
| HDL denotes high density lipoprotein cholesterol; LDL denotes low density lipoprotein cholesterol; IGF-1 denotes insulin-like growth factor 1; hsCRP denotes high sensitive C reactive protein. | | | | | | | |

| **Supplementary Table S2.** Baseline characteristics of participants stratified by sarcopenia defined by muscle specific strength, grip strength and muscle quality index | | | | | | | | | |
| --- | --- | --- | --- | --- | --- | --- | --- | --- | --- |
| **Characteristics: data show mean ± standard deviation or number (%)** | **MSS-sarcopenia** | | | **Sarcopenia** | | | **MQI-sarcopenia** | | |
|  | MSS-sarcopenia (20) | none  MSS-sarcopenia (1589) | *p*-value | sarcopenia (66) | none  sarcopenia (1543) | *p*-value | MQI-sarcopenia (32) | none  MQI-sarcopenia (1577) | *p*-value |
| **Demographic & lifestyle factors** |  |  |  |  |  |  |  |  |  |
| Age(years) | 65.6± 7.2 | 64.6± 6.9 | 0.564 | 62.9±6.3 | 64.7±7.0 | 0.023 | 64.4±7.1 | 64.7±6.9 | 0.862 |
| Male | 9(45.0) | 804(50.6) | 0.659 | 29(43.9) | 784(50.8) | 0.315 | 15(46.9) | 798(50.6) | 0.723 |
| Education level |  |  | 0.764 |  |  | 0.013 |  |  | 0.567 |
| Below elementary school | 5(25.0) | 390(24.5) |  | 7(10.6) | 388(25.2) |  | 6(18.8) | 389(24.7) |  |
| Junior high school | 2(10.0) | 253(15.9) |  | 9(13.6) | 246(15.9) |  | 4(12.5) | 251(15.9) |  |
| Above high school | 13(65.0) | 946(59.5) |  | 50(75.8) | 909(58.9) |  | 22(68.8) | 937(59.4) |  |
| Current alcohol | 2(10.0) | 385(24.2) | 0.189 | 12(18.2) | 375(24.3) | 0.304 | 6(18.8) | 381(24.2) | 0.676 |
| Current tobacco smoker | 1(5.0) | 141(8.9) | 1 | 6(9.1) | 136(8.8) | 0.827 | 3(9.4) | 139(8.8) | 0.757 |
| Body mass index(kg/m^2^) | 22.2±2.3 | 24.4±3.3 | <0.001 | 21.0±2.3 | 24.5±3.2 | <0.001 | 21.4±2.4 | 24.4±3.2 | <0.001 |
| **Medical history** |  |  |  |  |  |  |  |  |  |
| Hypertension | 6(30.0) | 505(31.8) | 1 | 12(18.2) | 499(32.3) | 0.015 | 8(25.0) | 503(31.90) | 0.450 |
| Diabetes | 1(5.0) | 231(14.5) | 0.342 | 5(7.6) | 227(14.7) | 0.149 | 2(6.3) | 230(14.6) | 0.304 |
| Coronary artery disease | 2(10.0) | 69(4.3) | 0.220 | 4(6.1) | 67(4.3) | 0.532 | 2(6.3) | 69(4.4) | 0.649 |
| Stroke | 0 | 16(1.0) | 1 | 0 | 16(1.0) | 1 | 0 | 16(1.0) | 1 |
| COPD | 0 | 7(0.4) | 1 | 0 | 7(0.5) | 1 | 0 | 7(0.4) | 1 |
| Chronic kidney disease | 3(0.9) | 6(0.5) | 0.394 | 0 | 9(0.6) | 1 | 0 | 9(0.6) | 1 |
| Charlson comorbidity index | 0.2±0.4 | 0.2±0.5 | 0.471 | 0.2±0.4 | 0.2±0.5 | 0.392 | 0.2±0.4 | 0.2±0.5 | 0.498 |
| **Functional assessments** |  |  |  |  |  |  |  |  |  |
| Mini Mental State Examination | 27.7±3.0 | 27.7±2.8 | 0.972 | 28.2±2.2 | 27.6±2.9 | 0.041 | 27.8±2.5 | 27.7±2.8 | 0.703 |
| Mini-Nutrition Assessment | 25.6±2.2 | 27.3±1.7 | 0.002 | 25.8±2.1 | 27.4±1.7 | <0.001 | 25.7±2.3 | 27.4±1.9 | <0.001 |
| CESD | 1.8±4.1 | 1.3±3.6 | 0.639 | 2.0±4.6 | 1.3±3.5 | 0.250 | 2.7±6.1 | 1.3±3.5 | 0.202 |
| SMAF-ADL | 0 | -0.01±0.25 | 0.032 | 0 | -0.01±0.25 | 0.033 | 0 | -0.01±0.25 | 0.033 |
| SMAF-IADL | -0.15±0.67 | -0.06±0.70 | 0.541 | -0.05±0.37 | -0.06±0.71 | 0.803 | -0.09±0.53 | -0.06±0.70 | 0.698 |
| **Muscle health parameters** |  |  |  |  |  |  |  |  |  |
| Muscle-specific strength | 10.3±0.9 | 13.4± 2.8 | 0.002 | 12.6± 2.1 | 13.4±2.8 | 0.001 | 11.2±1.4 | 13.5±2.8 | <0.001 |
| Grip strength(kg) | 22.4±8.0 | 31.0±8.9 | <0.001 | 23.1±6.5 | 31.2±8.9 | <0.001 | 21.5±6.2 | 31.0±8.9 | <0.001 |
| Skeletal muscle index(kg/m^2^) | 6.4±6.0 | 7.3±1.2 | 0.003 | 6.3±1.0 | 7.3±1.2 | <0.001 | 6.4±1.0 | 7.3±1.2 | <0.001 |
| Dominant hand muscle mass(kg) | 2.2±0.6 | 2.3±0.7 | 0.201 | 1.9±0.5 | 2.4±0.7 | <0.001 | 1.9±0.5 | 2.4±0.7 | <0.001 |
| Impaired physical performance | 12(60.0) | 515(32.4) | 0.014 | 27(40.9) | 500(32.4) | 0.180 | 15(46.9) | 512(32.5) | 0.090 |

MSS-sarcopenia denotes sarcopenia defined by muscle specific strength; MQI-sarcopenia denotes sarcopenia defined by muscle quality index;CESD denotes Center for Epidemiological Studies Depression Scale; SMAF denotes functional autonomy measurement system; ADL denotes activities of daily living; IADL denotes instrumental activities of daily living

| **Supplementary Table S3.** Associations of muscle-specific strength, grip strength, muscle quality index, and sarcopenia with impaired physical performance. | | | | | | | | | |
| --- | --- | --- | --- | --- | --- | --- | --- | --- | --- |
|  | model1 | | | model2 | | | model3 | | |
|  | OR | 95%CI | p-value | OR | 95%CI | p-value | OR | 95%CI | p-value |
| **Low muscle-specific strength** | 1.86 | (1.41 2.44) | <0.001 | 1.49 | (1.12 1.99) | 0.007 | 1.49 | (1.11 1.99) | 0.008 |
| **Low grip strength** | 1.13 | (0.83 1.53) | 0.442 | 0.90 | (0.65 1.25) | 0.518 | 0.90 | (0.65 1.25) | 0.534 |
| **Low muscle quality index** | 1.57 | (1.19 2.06) | 0.001 | 1.30 | (0.97 1.73) | 0.082 | 1.29 | (0.96 1.73) | 0.089 |
| **MSS-sarcopenia** | 2.61 | (1.08 6.31) | 0.033 | 2.82 | (1.12 7.12) | 0.028 | 3.31 | (1.26 8.74) | 0.015 |
| **sarcopenia(traditional)** | 0.75 | (0.42 1.35) | 0.334 | 0.99 | (0.54 1.82) | 0.975 | 1.07 | (0.54 2.12) | 0.854 |
| **MQI-sarcopenia** | 1.56 | (0.76 3.22) | 0.228 | 1.82 | (0.85 3.87) | 0.122 | 2.11 | (0.93 4.76) | 0.073 |
| Model 1 adjusted for age, sex, and education; Model 2 adjusted for model 1 plus baseline physical performance, Charlson comorbidity index and Mini-Mental State Examination; Model 3 adjusted for model 2 plus low relative appendicular skeletal muscle mass  Three definitions of sarcopenia model 1 adjusted crude model; model 2 for model 1 plus age, sex and education; model 3 for model 2 plus Charlson comorbidity index and, Mini Nutritional Assessment, SMAF-ADL and low relative appendicular skeletal muscle mass.  MSS denotes muscle-specific strength. MQI denotes muscle quality index. | | | | | | | | | |

| **Supplementary Table S4.** Muscle-specific strength and impaired physical performance stratified by age, sex and chronic conditions | | | | | | | |  | |  | |  |
| --- | --- | --- | --- | --- | --- | --- | --- | --- | --- | --- | --- | --- |
|  | model1 | | | model2 | | | model3 | | | | | |
|  | OR | 95%CI | p-value | OR | 95%CI | p-value | OR | | 95%CI | | p-value | |
| **Sex** |  |  |  |  |  |  |  | |  | |  | |
| Male | 2.02 | (1.37 2.97) | <0.001 | 1.64 | (1.09 2.46) | 0.017 | 1.64 | | (1.09 2.47) | | 0.018 | |
| Female | 1.71 | (1.17 2.51) | 0.006 | 1.36 | (0.90 2.06) | 0.144 | 1.36 | | (0.90 2.07) | | 0.148 | |
| **Age** |  |  |  |  |  |  |  | |  | |  | |
| < 65 years olds | 1.54 | (1.04 2.27) | 0.030 | 1.30 | (0.86 1.95) | 0.218 | 1.24 | | (0.82 1.88) | | 0.312 | |
| >= 65years olds | 2.27 | (1.55 3.32) | <0.001 | 1.71 | (1.13 2.58) | 0.011 | 1.80 | | (1.18 2.74) | | 0.006 | |
| **Chronic conditions.** |  |  |  |  |  |  |  | |  | |  | |
| Without diabetes^a^ | 1.82 | (1.35 2.48) | <0.001 | 1.50 | (1.09 2.07) | 0.014 | 1.52 | | (1.10 2.11) | | 0.011 | |
| Diabetes^a^ | 1.90 | (1.02 3.53) | 0.043 | 1.60 | (0.81 3.16) | 0.181 | 1.36 | | (0.67 2.73) | | 0.395 | |
| Without hypertension^b^ | 1.80 | (1.26 2.58) | 0.001 | 1.45 | (0.97 2.12) | 0.059 | 1.47 | | (1.00 2.16) | | 0.053 | |
| Hypertension^b^ | 1.78 | (1.17 2.72) | 0.008 | 1.44 | (0.91 2.27) | 0.117 | 1.40 | | (0.88 2.21) | | 0.153 | |
| Model 1 adjusted for age, sex, and education; Model 2 adjusted for model 1 plus baseline physical performance, Charlson comorbidity index and Mini-Mental State Examination; Model 3 adjusted for model 2 plus low relative appendicular skeletal muscle mass, and medications ( oral antidiabetic drugs or insulin^a^, and hypotensive drugs^b^). | | | | | | | | | | | | |

| **Supplementary Table S5.** Baseline biomarker profile stratified by muscle specific strength | | |  |
| --- | --- | --- | --- |
|  | Low muscle-specific strength  (n=320) | Normal muscle-specific strength  (n=1289) | *p*-value |
| **Aging-related biomarker** |  |  |  |
| IGF 1 (ng/mL) | 115.7±37.8 | 118.3±39.6 | 0.290 |
| Vitamin D 25-OH (ng/mL) | 25.6±7.9 | 25.8±7.8 | 0.614 |
| **Cardiometabolic biomarker** |  |  |  |
| Fasting glucose (mg/dL) | 101.0±27.3 | 95.0±18.7 | <0.001 |
| HbA1C (%) | 6.0±0.9 | 5.8±0.7 | <0.001 |
| HOMA-IR | 2.6±2.2 | 1.8±1.4 | <0.001 |
| HOMA-β | 111.3±78.6 | 94.1±66.1 | <0.001 |
| Total cholesterol (mg/dL) | 189.4±34.2 | 194.7±36.8 | 0.015 |
| Triglyceride (mg/dL) | 127.9±90.1 | 112.5±58.2 | 0.004 |
| High density lipoprotein (mg/dL) | 54.5±15.8 | 57.6±15.5 | 0.002 |
| Low density lipoprotein (mg/dL) | 109.3±31.4 | 114.6±32.5 | 0.008 |
| **Inflammation related biomarker** |  |  |  |
| High sensitivity CRP (mg/dL) | 0.19±0.34 | 0.16±0.48 | 0.089 |
| NLR | 1.9±0.9 | 1.9±0.9 | 0.947 |
| PLR | 8.2±3.5 | 8.0±3.9 | 0.404 |
| Homocysteine (μmol/L) | 14.2±5.9 | 13.6±4.8 | 0.104 |
| IGF-1 denotes insulin-like growth factor 1; HbA1c denotes glycated hemoglobin; HOMA-IR denotes Homeostatic Model Assessment for Insulin Resistance; HOMA-β denotes Homeostatic Model Assessment of β-cell function; CRP denotes C reactive protein. | | | |

| **Supplementary Table S6.** Multinomial logistic regressions explore associations between muscle-specific strength and biomarkers | | | | | | | | | | | |
| --- | --- | --- | --- | --- | --- | --- | --- | --- | --- | --- | --- |
|  | Model1 | | |  | Model2 | | |  | Model3 | | |
|  | OR | 95%CI | p-value |  | OR | 95%CI | p-value |  | OR | 95%CI | p-value |
| **Aging-related biomarker** |  |  |  |  |  |  |  |  |  |  |  |
| IGF 1(medium vs. low) | 0.90 | (0.67 1.23) | 0.514 |  | 0.94 | (0.69 1.28) | 0.694 |  | 0.96 | (0.70 1.31) | 0.793 |
| IGF 1(high vs. low) | 0.88 | (0.64 1.21) | 0.442 |  | 0.88 | (0.65 1.24) | 0.523 |  | 0.89 | (0.64 1.23) | 0.463 |
| Vitamin D 25-OH^a^ (medium vs. low) | 0.85 | (0.63 1.16) | 0.306 |  | 0.87 | (0.64 1.18) | 0.370 |  | 0.89 | (0.65 1.22) | 0.466 |
| Vitamin D 25-OH^a^ (high vs. low) | 0.88 | (0.64 1.20) | 0.415 |  | 0.88 | (0.64 1.20) | 0.415 |  | 0.86 | (0.62 1.18) | 0.352 |
| **Cardiometabolic biomarker** |  |  |  |  |  |  |  |  |  |  |  |
| Fasting glucose^b^ (medium vs. low) | 1.31 | (0.95 1.82) | 0.099 |  | 1.26 | (0.91 1.75) | 0.168 |  | 1.22 | (0.88 1.70) | 0.239 |
| Fasting glucose^b^ (high vs. low) | 1.80 | (1.33 2.44) | <0.001 |  | 1.76 | (1.29 2.42) | <0.001 |  | 1.55 | (1.11 2.16) | 0.011 |
| HbA1C^b^ ((medium vs. low) | 1.19 | (0.86 1.65) | 0.286 |  | 1.20 | (0.86 1.67) | 0.274 |  | 1.20 | (0.86 1.68) | 0.282 |
| HbA1C^b^ (high vs. low) | 1.78 | (1.33 2.38) | <0.001 |  | 1.80 | (1.32 2.46) | <0.001 |  | 1.66 | (1.18 2.33) | 0.003 |
| HOMA-IR^b^ (medium vs. low) | 1.46 | (1.03 2.07) | 0.032 |  | 1.41 | (0.99 2.01) | 0.055 |  | 1.31 | (0.92 1.86) | 0.142 |
| HOMA-IR^b^ (high vs. low) | 3.29 | (2.39 4.54) | <0.001 |  | 3.10 | (2.24 4.30) | <0.001 |  | 2.71 | (1.94 3.79) | <0.001 |
| HOMA-β^b^ (medium vs. low) | 0.99 | (0.71 1.37) | 0.952 |  | 1.02 | (0.73 1.43) | 0.891 |  | 1.07 | (0.76 1.50) | 0.709 |
| HOMA-β^b^ (high vs. low) | 1.91 | (1.42 2.59) | <0.001 |  | 1.97 | (1.45 2.69) | <0.001 |  | 1.94 | (1.41 2.67) | <0.001 |
| Total cholesterol^c^ (medium vs. low) | 0.92 | (0.69 1.23) | 0.570 |  | 0.92 | (0.68 1.25) | 0.590 |  | 0.95 | (0.70 1.30) | 0.766 |
| Total cholesterol^c^ (high vs. low) | 0.66 | (0.48 0.90) | 0.010 |  | 0.66 | (0.48 0.92) | 0.014 |  | 0.70 | (0.49 0.98) | 0.040 |
| Triglyceride^c^ (medium vs. low) | 1.23 | (0.91 1.68) | 0.180 |  | 1.24 | (0.91 1.69) | 0.177 |  | 1.18 | (0.86 1.62) | 0.306 |
| Triglyceride^c^ (high vs. low) | 1.28 | (0.94 1.74) | 0.123 |  | 1.22 | (0.89 1.67) | 0.209 |  | 1.17 | (0.85 1.60) | 0.346 |
| HDL^c^ (medium vs. low) | 0.62 | (0.46 0.83) | 0.002 |  | 0.65 | (0.48 0.88) | 0.006 |  | 0.68 | (0.50 0.92) | 0.012 |
| HDL^c^ (high vs. low) | 0.53 | (0.39 0.74) | <0.001 |  | 0.57 | (0.41 0.80) | 0.001 |  | 0.65 | (0.46 0.91) | 0.012 |
| LDL^c^ (medium vs. low) | 1.04 | (0.77 1.40) | 0.803 |  | 1.05 | (0.77 1.42) | 0.767 |  | 1.09 | (0.80 1.49) | 0.584 |
| LDL^c^ (high vs. low) | 0.77 | (0.56 1.05) | 0.097 |  | 0.79 | (0.57 1.09) | 0.143 |  | 0.79 | (0.57 1.12) | 0.197 |
| **Inflammation related biomarker** |  |  |  |  |  |  |  |  |  |  |  |
| HsCRP(medium vs. low) | 1.19 | (0.86 1.64) | 0.303 |  | 1.23 | (0.89 1.71) | 0.210 |  | 1.18 | (0.85 1.65) | 0.315 |
| HsCRP(high vs. low) | 1.80 | (1.32 2.44) | <0.001 |  | 1.84 | (1.34 2.51) | <0.001 |  | 1.75 | (1.28 2.39) | 0.001 |
| NLR(medium vs. low) | 1.10 | (0.81 1.50) | 0.533 |  | 1.09 | (0.80 1.48) | 0.595 |  | 1.08 | (0.79 1.47) | 0.651 |
| NLR(high vs. low) | 1.06 | (0.77 1.44) | 0.736 |  | 1.00 | (0.73 1.38) | 0.983 |  | 1.00 | (0.73 1.38) | 0.990 |
| PLR(low vs. low) | 1.04 | (0.77 1.42) | 0.796 |  | 1.06 | (0.78 1.45) | 0.708 |  | 1.08 | (0.79 1.48) | 0.637 |
| PLR(high vs. low) | 1.15 | (0.85 1.55) | 0.377 |  | 1.15 | (0.85 1.57) | 0.367 |  | 1.19 | (0.87 1.62) | 0.276 |
| Homocysteine^d^ (low vs. low) | 1.04 | (0.76 1.44) | 0.792 |  | 1.02 | (0.74 1.41) | 0.913 |  | 0.98 | (0.71 1.35) | 0.889 |
| Homocysteine^d^ (high vs. low) | 1.21 | (0.86 1.69) | 0.274 |  | 1.12 | (0.80 1.58) | 0.517 |  | 1.06 | (0.75 1.49) | 0.747 |
| IGF-1 denotes insulin-like growth factor 1; HbA1c denotes glycated hemoglobin; HOMA-IR denotes Homeostatic Model Assessment for Insulin Resistance; HDL denotes high density lipoprotein cholesterol; HOMA-β denotes Homeostatic Model Assessment of β-cell function; LDL denotes low density lipoprotein cholesterol; hsCRP denotes high sensitivity C reactive protein; NLR denotes neutrophil lymphocyte ratio; PLR denotes platelet lymphocyte ratio.  IGF-1 Low: < 99.4 ng/mL, Medium: 99.4 – 128 ng/mL, High: > 128 ng/mL;Vitamin D 25-OH Low: < 21.8 ng/mL,Medium: 21.8 – 28.2 ng/mL ,High: > 28.2 ng/mL; fasting glucose AC Low: < 87 mg/dL,Medium: 87 – 96 mg/dL, High: > 96 mg/dL; HbA1C Low: < 5.5%,Medium: 5.5% – 5.8% ,High: > 5.8%;HOMA-IR Low: < 1.15,Medium: 1.15 – 1.97 ,High: > 1.97;Total Cholesterol Low: < 177 mg/dL,Medium: 177 – 207.4 mg/dL,High: > 207.4 mg/dL; Triglycerides Low: < 82 mg/dL,Medium: 82 – 120 mg/dL ,High: > 120 mg/dL;HDL Low: < 48 mg/dL,Medium: 48 – 61 mg/dL,High: > 61 mg/dL; LDL Low: < 98 mg/dL;Medium: 98 – 126 mg/dL,High: > 126 mg/dL; HsCRP Low: < 0.021 mg/L,Medium: 0.021 – 0.092 mg/L (inclusive),High: > 0.092 mg/L;Neutrophil-to-Lymphocyte Ratio (NLR) Low: < 1.47,Medium: 1.47 – 2.05 (inclusive),High: > 2.05;Platelet-to-Lymphocyte Ratio (PLR) Low: < 6.32,Medium: 6.32 – 8.53 (inclusive),High: > 8.53;Homocysteine Low: < 11.4 µmol/L,Medium: 11.4 – 14.6 µmol/L (inclusive),High: > 14.6 µmol/L；HOMA-β Low:<64.6, Medium:64.6-105.2, High:>105.2. Model 1 adjusted for age, sex, and education; Model 2 adjusted for model 1 plus baseline physical performance, Charlson cormobidity index and Mini-Mental State Examination; Model 3 adjusted for model 2 plus low relative appendicular skeletal muscle mass, and medications (vitamin D^a^, glucose-lowering drugs^b^, lipid-lowering drugs^c^, and vitamin B supplements^d^) | | | | | | | | | | | |


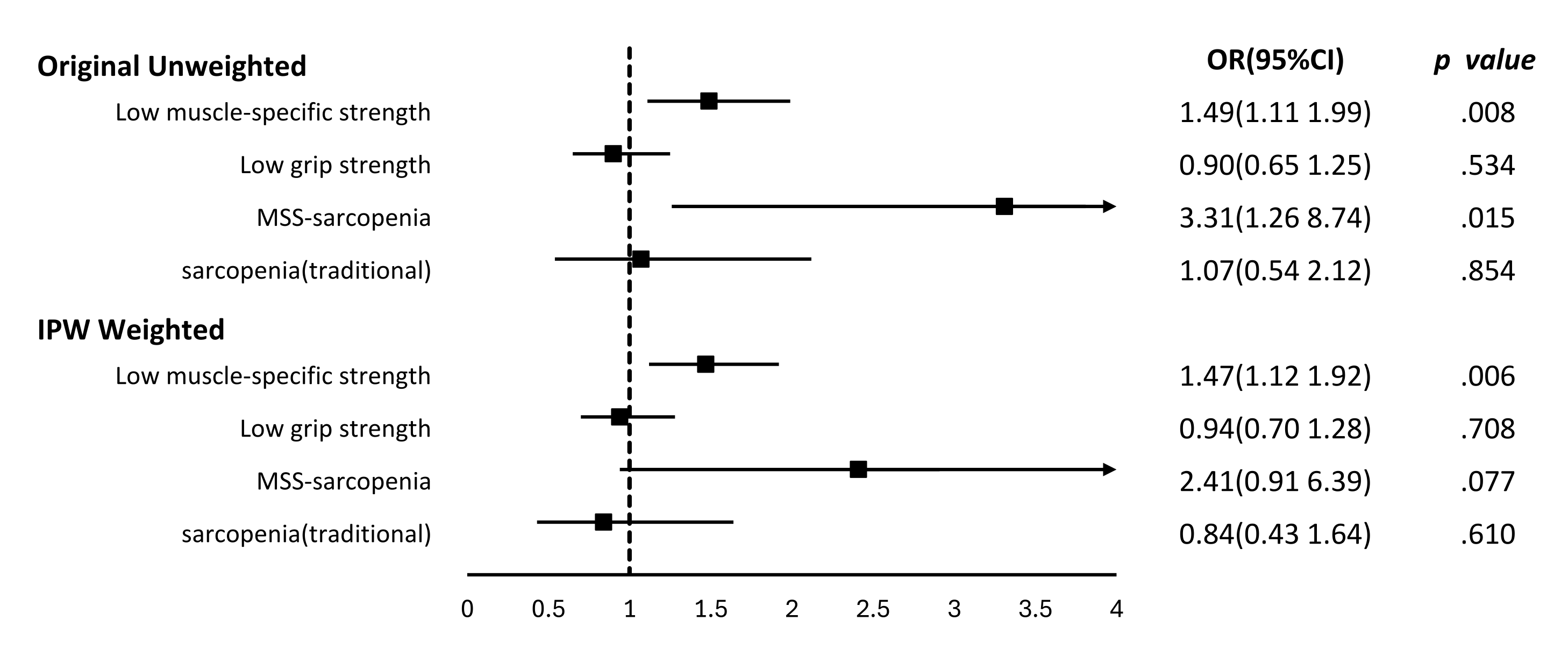
**Supplementary Figure S1.** Sensitivity analysis using inverse probability weighting to account for missing follow-up data

Logistic regression analysis adjusted for age, sex, education, baseline physical performance, Charlson comorbidity index, Mini-Mental State Examination, and low relative appendicular skeletal muscle mass.

**Supplementary Figure S2.** Associations of MSS, sarcopenic obesity, and MSS-sarcopenic obesity with impaired physical performance.


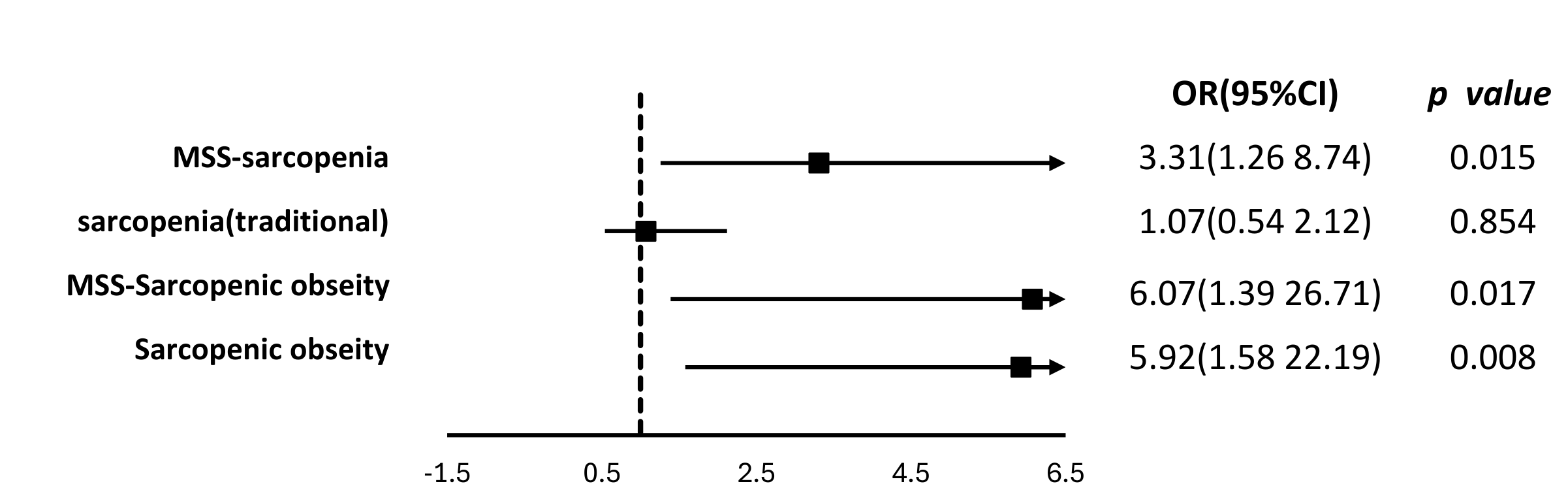


Logistic regression analysis adjusted for age, sex, education, baseline physical performance, Charlson comorbidity index, Mini-Mental State Examination, and low relative appendicular skeletal muscle mass.
